# Osteoblast Attachment on Titanium Coated with Hydroxyapatite by Atomic Layer Deposition

**DOI:** 10.3390/biom12050654

**Published:** 2022-04-29

**Authors:** Elina Kylmäoja, Jani Holopainen, Faleh Abushahba, Mikko Ritala, Juha Tuukkanen

**Affiliations:** 1 Department of Anatomy and Cell Biology, Institute of Cancer Research and Translational Medicine, Medical Research Center, University of Oulu, P.O. Box 5000, 90014 Oulu, Finland; juha.tuukkanen@oulu.fi; 2 Department of Chemistry, University of Helsinki, P.O. Box 55, 00014 Helsinki, Finland; jani.h.holopainen@outlook.com (J.H.); mikko.ritala@helsinki.fi (M.R.); 3 Department of Prosthetic Dentistry and Stomatognathic Physiology, Institute of Dentistry, University of Turku, 20520 Turku, Finland; faleh.a.abushahba@utu.fi

**Keywords:** atomic layer deposition, hydroxyapatite, titanium, osteoblast, MC3T3

## Abstract

Background: The increasing demand for bone implants with improved osseointegration properties has prompted researchers to develop various coating types for metal implants. Atomic layer deposition (ALD) is a method for producing nanoscale coatings conformally on complex three-dimensional surfaces. We have prepared hydroxyapatite (HA) coating on titanium (Ti) substrate with the ALD method and analyzed the biocompatibility of this coating in terms of cell adhesion and viability. Methods: HA coatings were prepared on Ti substrates by depositing CaCO_3_ films by ALD and converting them to HA by wet treatment in dilute phosphate solution. MC3T3-E1 preosteoblasts were cultured on ALD-HA, glass slides and bovine bone slices. ALD-HA and glass slides were either coated or non-coated with fibronectin. After 48h culture, cells were imaged with scanning electron microscopy (SEM) and analyzed by vinculin antibody staining for focal adhesion localization. An 3-[4,5-dimethylthiazol-2-yl]-2,5-diphenyl tetrazolium bromide (MTT) test was performed to study cell viability. Results: Vinculin staining revealed similar focal adhesion-like structures on ALD-HA as on glass slides and bone, albeit on ALD-HA and bone the structures were thinner compared to glass slides. This might be due to thin and broad focal adhesions on complex three-dimensional surfaces of ALD-HA and bone. The MTT test showed comparable cell viability on ALD-HA, glass slides and bone. Conclusion: ALD-HA coating was shown to be biocompatible in regard to cell adhesion and viability. This leads to new opportunities in developing improved implant coatings for better osseointegration and implant survival.

## 1. Introduction

Global ageing and diverse accidents occurring in free time activities have caused an increased demand for implantable devices for repairing human tissues. For bone implants, the attachment of the implant to the bone tissue along with new bone formation, termed osseointegration, is extremely important. Failed osseointegration, biomechanical changes and micromotions over time can lead to implant loosening, causing a need for revision surgery [1,2].

Bone implants are commonly made of metals, ceramics or polymers and often their biocompatibility is enhanced by surface modifications. Cellular attachment is the first and most indispensable event in osseointegration and it affects the overall survival of the implant. Before the cells attach to the implant surface, it becomes coated with endogenous proteins. To avoid random coating with various proteins present in the body, and thus improve cellular attachment, several extracellular matrix proteins, such as collagen, laminin and fibronectin, have been utilized to synthetically coat the implants before implantation [3,4].

Titanium (Ti) and Ti-based alloys have for long been used to treat hard-tissue injuries, as Ti has many advantages over other metal implants. For instance, Ti is non-toxic, resistant to corrosion and has good mechanical properties [2,5]. Hydroxyapatite (HA; Ca_10_(PO_4_)_6_(OH)_2_) coating of Ti implants is a surface modification that has been shown to improve osseointegration [2,5,6,7,8,9,10,11]. As the natural bone and teeth mineral, HA has been found to be advantageous for coating bone implants [12]. Bone can directly grow into HA coating, creating a stable connection to the implant [11,13,14,15,16]. In addition to better osseointegration, the HA coating prevents the release of metal particles from the implant [17,18,19].

Besides being beneficial for bone growth within the implant, the HA coating seems to be a target for degradation over time [20,21,22,23]. Interestingly, the degradation does not affect the fixation of the implant, as in many cases the HA has been totally replaced by new bone [20,21]. The mechanism behind this phenomenon is osteoclast-mediated bone resorption and subsequent bone remodeling producing the new bone [20,22,24,25,26,27]. In fact, some studies provide evidence that the initial osteoclast activity is a prerequisite for bone formation around the implant [28,29,30,31]. An important mediator of osteoclast differentiation and resorption is the vast macrophage population around the implant, resulting from the insertion of a foreign body into the tissue. These macrophages or foreign body cells can either produce osteoclast activating molecules or serve as precursors for differentiating osteoclasts [32,33,34,35,36].

HA coatings can be made by various methods, such as the sol-gel, plasma spraying, laser ablation and sputter coating. However, these methods are not always ideal since they might need a very high processing temperature and tend to produce coatings with unfavorable properties such as brittleness and flaking or cracking [37,38,39]. In addition, many of these methods are expensive and cannot be used to coat complex-shaped implants with a uniform coating thickness [37]. One method to overcome these problems is atomic layer deposition (ALD), which can coat complicated three-dimensional surfaces with a thin conformal layer. In the ALD process alternating pulses of gaseous precursors deposit a thin layer on a substrate via self-limiting reactions. The coating can be made layer by layer in nanometer scale, which is difficult with other methods [40].

Only a few studies have been conducted concerning cellular activities on surfaces made with the ALD technique. Recent studies utilized ALD in preparing TiO_2_ films and showed that the coating improved human osteoblast C-12720 [41], MG-63 [42] and murine osteoblast lineage MC3T3 cell [43] adhesion, proliferation and differentiation [44], but inhibited these activities in fibroblasts [41] and had an inhibitory effect on osteoclast invasion [43]. Motola et al. [45] coated Ti and Ti nanotube surfaces with an additional ALD TiO_2_ coating and reported increased WI-38 fibroblast and MG-63 osteoblast growth on the coated surfaces. Zemtsova et al. [46] produced a titano-organic coating from TiCl_4_ and propargyl alcohol with ALD and showed increased differentiation of MC3T3 cells and better osseointegration in a rabbit model. Liang et al. [47] observed enhanced HA formation in simulated body fluid on ALD alumina (Al_2_O_3_) and TiO_2_ coatings, and demonstrated that NIH/3T3 fibroblasts attached to the coatings. Radtke et al. [48] showed that murine L929 fibroblasts attached to Ti6Al4V samples coated with Ti nanotubes and ALD-HA, and that cell proliferation was increased on the coated surfaces compared to non-coated surface and the surface without the Ti nanotube layer. In addition, thin films of zirconia produced by ALD have been shown to increase the viability and differentiation of MC3T3 cells [49,50]. A recent review article by Astaneh et al. [51] summarized the physical and clinical properties of ALD coated dental materials.

We have utilized ALD in preparing a nanocrystalline HA coating on Ti substrate by converting ALD-CaCO_3_ to HA by a chemical treatment in dilute phosphate solution [52]. We have also recently tested the mechanical properties of different versions of this coating by tensile adhesion and scratching tests [53]. The coatings were found to be intact and suitable for further investigation, such as cell attachment and biocompatibility. Although we showed in the first study that human bone marrow-derived cells can be cultured on this nanocrystalline HA coating, we did not characterize cell adhesion thoroughly with cell biological methods.

The purpose of this study was to characterize cell attachment on the nanocrystalline HA coating generated on Ti samples with the ALD method. We were interested in whether the osteoblastic cell line MC3T3-E1 cells would attach to the surface with similar focal adhesions as they attach to glass slides and bone, and whether the cells would have normal morphology. In addition, we tested whether fibronectin (FN) coating of the samples affects cell adhesion. The hypothesis that the coating would be biocompatible concerning cellular attachment would aid in developing these thin HA coatings with beneficial properties.

## 2. Materials and Methods

### 2.1. Preparation of Nanocrystalline HA Coating (ALD-HA) on Ti Substrates with ALD Method

The HA coatings were made on Ti substrates as described in [52,53]. The substrate for ALD HA coating was a 1 mm thick titanium sheet (Grade 2, ASTM B265 specification, William Gregor Ltd., London, UK). The ALD coating was started by depositing a thin film of CaCO_3_ in a F-120 ALD reactor (ASM Microchemistry Ltd., Helsinki, Finland) with nitrogen carrier and purging gas. The CaCO_3_ films were deposited using the Ca(thd)_2_-O_3_ process previously reported in the literature [54]. Ca(thd)_2_ (Volatec Oy, Porvoo, Finland) was evaporated at 188 °C and O_3_ was generated from O_2_ (99.9999%) with a Wedeco Ozomatic Modular 4 HC Lab ozone generator. Pulses and purges of 1 s were used for all precursors. The depositions were conducted at 250 °C. Conversion of CaCO_3_ to HA was achieved by using 0.2 M (NH_4_)_2_HPO_4_ (Merck, 99%) solution at 95 °C. After conversion, the samples were rinsed with deionized water and blown dry with compressed air. Samples were produced with 4000 cycles. A manual plate cutter (Bernardo PTS 1050 S Manual disc cutter, Linz, Austria) was used for cutting the ALD coated titanium plates. The Ti plate was firmly placed in a disc pressing to keep it in place during the cutting process. Then the plate was cut to produce 1 cm^2^ square-shaped size discs. Before cell culture, the samples were soaked in 70% ethanol for 10 min and air-dried.

### 2.2. MC3T3-E1 Cell Culture

Osteoblastic MC3T3-E1 cells were obtained from Merck Life Science Oy, Darmstadt, Germany and cultured in α-MEM (Gibco; Thermo Fisher Scientific, Waltham, MA, USA) without ascorbic acid but containing 10% fetal bovine serum (FBS) (Biowest, Riverside, MO, USA), 100 IU/mL penicillin and 100 µg/mL streptomycin and 24 mM Hepes buffer (Sigma-Aldrich, St. Louis, MO, USA) at +37 °C (5% CO_2_, 95% air). Fibronectin coating of cover glasses and ALD-HA was performed using phosphate buffered saline (PBS) with 10 ng/mL FN (Sigma-Aldrich). A volume of 200 µL of the dilution was incubated on the samples for 2 h at +37 °C, after which the samples were dried. Before cell seeding, all samples were soaked in cell culture medium for 10 min. For culturing on cover glasses, bone slices or ALD-HA samples, 10,000 cells/cm^2^ were seeded on the samples in 24-well plates (Cellstar; Greiner Bio-One, Kremsmünster, Austria) and cultured for 48 h. Sonicated bovine cortical bone slices (0.28 cm^2^) were obtained from Lehenkari Consulting, Oulu, Finland.

### 2.3. Focal Adhesion Staining

The cells were fixed and permeabilized with 4% paraformaldehyde (PFA)-0.3% Triton X-100-PBS for 10 min and blocked with 0.2% bovine serum albumin (BSA) (Sigma-Aldrich) for 30 min at room temperature (RT). Focal adhesions were stained with 1:100 diluted monoclonal anti-vinculin (Nordic BioSite Oy, Helsinki, Finland) for 1 h at RT and secondary antibody goat anti-mouse Alexa 488 (2 mg/mL stock diluted 1:100 in PBS, Molecular Probes; Thermo Fisher Scientific) for 1 h at RT. The actin cytoskeleton was stained with TRITC-conjugated phalloidin (0.1 mg/mL stock diluted 1:100 in PBS; Sigma-Aldrich) for 20 min at +37 °C. Nuclei were stained with Hoechst 33258 (1 mg/mL stock diluted 1:800 in PBS; Sigma-Aldrich) for 10 min at RT. The cover glasses were mounted in ImmuMount (Thermo Fisher Scientific) and ALD-HA samples and bone slices were mounted in 70% glycerol-PBS. The cells were viewed with Leica TCS SP8 confocal with a DMI8 microscope (Leica, Wetzlar, Germany) using LAS X 3.5.2 acquisition software (Leica). The objective used was an HC PL APO CS2 63 x/1.40 Oil. Samples were imaged with 405, 499 and 551 nm solid-state lasers with emission windows at 410–494, 509–556 and 561–754 nm, respectively. The pinhole was set to Airy 1 and scan speed to 600 Hz. Images were acquired with 1.48 zoom (pixel size 0.059 µm). Maximum intensity projections (MIP) were created from the Z-stacks.

### 2.4. Field Emission Scanning Electron Microscopy (FESEM)

The ALD-HA samples were dehydrated in ascending ethanol series and dried with a critical point drying equipment K850 (Quorum Technologies, Lewes, UK). Samples were coated with 5 nm platinum by Q150T ES sputter coater (Quorum Technologies, Lewes, UK) and viewed with Sigma HD VP FE-SEM (Carl Zeiss Microscopy GmbH, Oberkochen, Germany). FESEM images were taken with 5.0 kV voltage.

### 2.5. Cell Morphology Measurement

The average aspect ratios (major axis/minor axis) of the cells (n ≥ 5) were measured from confocal microscopy images with QuPath bioimage analysis software, version 0.3.2 (University of Edinburgh, Edinburgh, UK) from the snapshot sent to Image-J, version 1.53i (NIH, Bethesda, MD, USA).

### 2.6. Cell viability Assay with MTT

After 48 h culture, the medium was removed, fresh medium with 0.5 mg/mL 3-[4,5-dimethylthiazol-2-yl]-2,5-diphenyl tetrazolium bromide (MTT; Sigma-Aldrich) dye was added to the wells and incubated at +37 °C for 4 h. Thereafter, the medium was replaced with an equal volume of dimethyl sulfoxide (DMSO; Merck, Germany) and mixed. Cell viability was assessed by measuring absorbances at wavelengths 550 and 650 nm (background) with Victor 2 multilabel counter (Perkin Elmer/Wallac, Turku, Finland). Cell viability on bone slices and ALD-HA samples was compared to cover glasses, which were treated as controls by setting their viability to 100%.

### 2.7. Statistical Analysis

All experiments were done with groups of n ≥ 3 and repeated with three independent samples. Statistical analyses were performed with SPSS version 26 (SPSS Inc., Chicago, IL, USA). The normality of the response variables was tested with the Shapiro–Wilk test and histogram visualization. Since the response variables were not normally distributed, statistical differences between the test groups were evaluated using the Kruskal–Wallis test, and a comparison between groups was performed using Mann–Whitney U-test. The graphical presentation of the results was created with OriginPro 9.7 software (OriginLab, Northampton, MA, USA). *p* < 0.05 was considered significant. Data are shown as mean ± SEM.

## 3. Results

### 3.1. MC3T3 Cells Attached to ALD-HA

**Figure 1:**
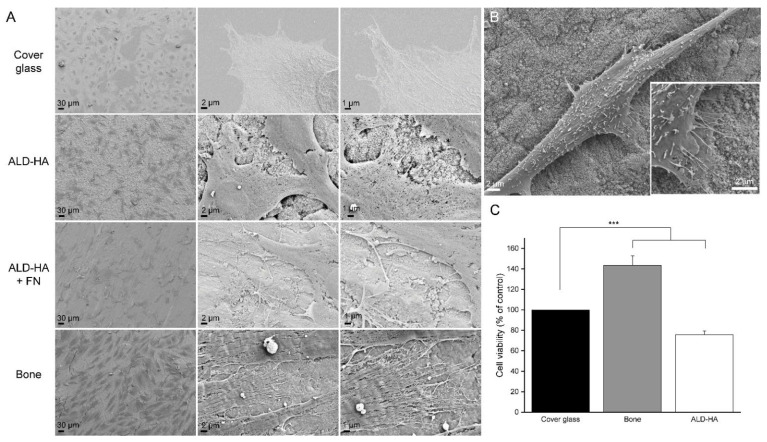
Morphology and viability of MC3T3-cells on cover glass, ALD-HA and bone. (**A**) FESEM images of MC3T3 cells cultured on cover glass, ALD-HA, FN-coated ALD-HA and bone (representative data from one culture). The cells were cultured for 48 h. The samples were evenly covered with cells, and the cells seemed to attach to the surface with focal adhesion-like structures. Magnification: 150× (left panel), 2500× (center panel) and 5000× (right panel). (**B**) Morphology of one MC3T3 cell cultured on ALD-HA. Magnification 2500×. (**C**) MTT test results of MC3T3 cells cultured on cover glass, bone slices and ALD-HA. MTT test was performed after 48 h cell culture on the samples. Cell viability on bone slices and ALD-HA samples was compared to cover glasses, which were treated as controls by setting their viability to 100%. The data is pooled from three independent cell cultures and shown as mean ± SEM. *** *p* <0.001.

MC3T3 cells were cultured on cover glasses, ALD-HA, FN-coated ALD-HA and bone slices for 48 h and imaged with FESEM. The cells on all samples were spread uniformly, their morphology was normal and the cells were attached to the surfaces (Figure 1A). On cover glasses, the cell morphology was flatter compared to ALD-HA and bone. On ALD-HA and bone, the cells seemed to attach to the surface with thin focal adhesion-like structures at the actin cytoskeleton protrusions or at the tips of long filopodia (Figure 1B). Concerning the cell morphology, the average aspect ratios (major axis/minor axis) of the cells were 1.4 ± 0.14 on cover glass, 2.3 ± 1.12 on ALD and 6.7 ± 2.33 on bone slice. The results indicate that the cell morphology on cover glass was relatively circular, whereas on ALD-HA and bone the morphology was more elongated.

### 3.2. MTT-Results Confirmed the Viability of Cells Cultured on ALD-HA

We tested whether the cell viability on ALD-HA was comparable to viability on cover glasses and bone. Cover glasses were used as a control, and the results showed that on bone slices the viability was significantly higher compared to cover glasses (*p* < 0.001). In contrast, on ALD-HA the viability was significantly lower (*p* < 0.001) (Figure 1B). The results show that there are viable cells on the ALD-HA samples. The lower viability percent compared to cover glasses and bone depicts that the cell number is lower on ALD-HA, as the MTT test values are directly proportional to the number of viable cells on the samples.

### 3.3. Thin Focal Adhesion-Like Structures Were Observed in MC3T3-Cells Cultured on ALD-HA

**Figure 2:**
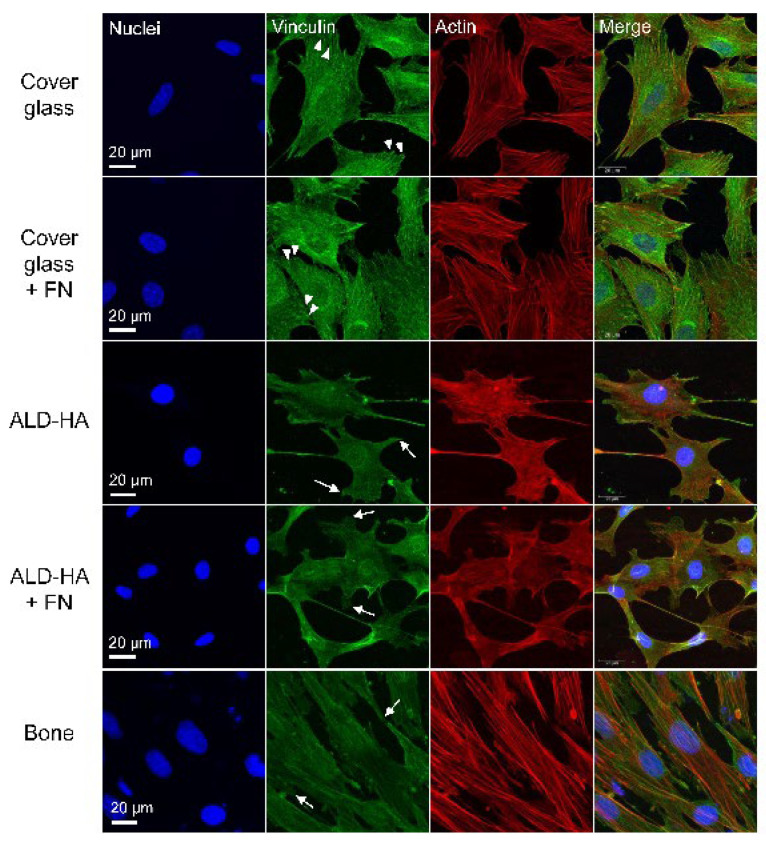
Focal adhesion localization in MC3T3 cells. Focal adhesion staining of MC3T3 cells on cover glass, ALD-HA and bone slice after 48h culture (representative data from one culture). Nuclei were stained with Hoechst 33258 (blue), focal adhesions with anti-vinculin (green) and actin cytoskeleton with TRITC-phalloidin (red). Images were taken with fluorescence microscope and 63× objective with 1.48 zoom, and maximum intensity projections were created from Z-stacks. Cover glasses and ALD-HA samples were either non-coated or coated with fibronectin (FN). Focal adhesion-like structures were present at the edges of the cells on all surfaces, although on ALD-HA and bone these structures were thinner (arrows) compared to thicker dot-like structures observed on cover glass (arrowheads). Fibronectin coating did not have an effect on the cell morphology or vinculin staining.

When MC3T3-E1 cells were cultured on cover glasses, the vinculin staining showed small dot-like structures on the edges of the cells representing the cell attachment with focal adhesions (Figure 2). In addition, constant cytoplasmic vinculin was observed. Coating the cover glass with FN did not affect the vinculin localization or cell morphology. When cells were cultured on ALD-HA or bone slices, the vinculin staining was slightly dimmer, but thin focal adhesion-like structures were observed on the edges of the cells. As with cover glasses, vinculin was also present in the cytoplasm, and the FN coating did not affect vinculin localization or cell morphology on cells cultured on ALD-HA. Thus, the FN coating did not seem to have remarkable benefits concerning cell adhesion. In regard to actin stress fiber staining, the cells on ALD-HA coated surfaces were lacking the stress fibers nearly completely, whereas on cover glass and bone the stress fibers were clearly distinguishable.

## 4. Discussion

We have previously shown that human bone marrow-derived cells can be cultured on nanocrystalline HA-coated titanium substrates prepared with the ALD method [52]. In addition, in our tests, the coating was stable [53], and the bone marrow-derived monocytes could fuse into multinuclear cells on the coating, as they do on natural bone slices [52].

Several studies have been made to investigate bone cell attachment to HA. For instance, it has been shown that rat bone marrow mesenchymal stem cell attachment, cell viability and ALP expression were higher on a polycaprolactone-polytetrahydrofuran-HA composite scaffold compared to the HA-deficient composite [55]. Other studies showed that HA coating on TiO_2_ nanotubes [56,57] and titanium disks [58] supports osteoblast lineage MC3T3 cell attachment, proliferation and differentiation. Polylactic acid (PLA)-HA composite films were also superior to neat PLA in the promotion of MC3T3-E1 cell attachment as well as in the induction of focal adhesions dose dependently [59]. Opposite results of the benefits of HA coating have also been observed, as Kobayashi et al. [60] showed that HA dispersed into a Ti-based composite inhibited MC3T3 adhesion and proliferation in a concentration-dependent manner.

This is the first study characterizing in detail the cell attachment on HA coating made with the ALD method. In addition to studying the cellular attachment to ALD-HA, we wanted to study if FN coating would improve the attachment of MC3T3 cells on the surface. The initial attachment to a surface occurs with a multiprotein complex, focal adhesion, including, among others, vinculin, talin and paxillin [61]. Immunofluorescence staining of these proteins can be used to localize focal adhesions. We were able to show thin focal adhesion-like structures based on vinculin immunofluorescence in MC3T3 cells cultured on ALD-HA coating, as well as on ALD-HA coating additionally treated with FN. The cell morphology and spreading were similar on both surfaces, indicating that the additional FN layer did not enhance the biocompatibility of the surface in relation to cell attachment. Our results are to some extent contrary to earlier studies, where better MC3T3 cell attachment [62], higher viability [63] and enhanced differentiation [64,65] on FN-coated titanium surfaces compared to bare titanium was observed. However, these studies were made with FN-coated titanium samples without the additional HA layer between the Ti and FN. Although Pramono et al. [65] showed the benefits of FN-coating for MC3T3 attachment, they did not detect differences in the cell morphology between the coated and non-coated surfaces, which supports our observation of the similar morphology on different surfaces. Further, in a continuation to the study by Pugdee et al., Yoshida et al. [66] noticed that FN-coating of Ti possibly enhances the initial adhesion, but not proliferation or activity of MC3T3 cells. The authors point out that FN is nevertheless produced in cell culture, and therefore the FN-coating might not offer remarkable benefits for cellular adhesion. In their study, the main changes in cell morphology were caused by mechanical treatment (sandblasting) of the Ti surface, but not the FN-coating. As mentioned in the previous paragraph, in the article by Kobayashi et al. [60], HA in Ti-composite plates inhibited MC3T3 adhesion and proliferation, but the coating of the plates with FN decreased the inhibitory effect of HA. Similar to the study by Yoshida et al. [66], the main changes in the cellular activities were caused by the manufacturing process (sintering temperature) of the samples instead of the FN coating [60]. Also, Noh et al. [67] showed that although FN improved adhesion of MC3T3 and monocyte-macrophage lineage Raw 264.7 cells on Ti disks, a more pronounced improvement in the adhesion was obtained by increasing the surface roughness. Concerning osseointegration properties of HA-FN coating, it was shown that an HA-FN-coated dental implant in a canine model did not improve the results compared to non-coated implants [68].

The surface roughness of Ti and its alloys is well known to affect cellular attachment and osseointegration of implants [69,70,71], and several studies have shown that a combination of micro- and nanoscale surface roughness leads to optimal results [42,72,73,74,75,76,77,78]. Several studies have examined the optimal surface roughness for MC3T3 adhesion and differentiation. Some studies have found that roughness in the micrometer range (0.5–2.5 µm) is preferable to smoother or rougher surfaces [63,79,80,81,82]. Iwaya et al. [83] reported no differences in MC3T3 cell proliferation and collagen production between Ti disks with a surface roughness from 0.34 µm to 2 µm. Still, this roughness range fits well to the aforementioned micrometer range that was found optimal in other studies. Regarding the nanometer range modifications, 100 nm roughness has been reported to inhibit MC3T3 attachment, spreading and differentiation compared to smooth Ti surface [84,85]. On the contrary, on Ti-alloy coated with Ti nanotubes and ALD-HA, a roughness of 135 nm was found to be optimal for fibroblast adhesion and proliferation compared to smoother surfaces [48]. In addition, combinations of micro- and nanoscale roughness have also been suggested to be favorable for MC3T3 adhesion and differentiation [46,86,87]. However, as Nobles et al. [71] and Wennerberg et al. [69] remark, the aforementioned studies have utilized a wide range of surface features and modification techniques, leading to difficulties in interpreting the results, i.e., whether they are caused by the surface characteristics or the techniques they were manufactured by.

Regarding the observation of the focal adhesion-like attachment of the cells on the ALD-HA coating, we did not detect increased vinculin expression on HA-coating, which was shown on calcium and phosphate ion-modified Ti-coating by Sunarso et al. [88]. In their study, vinculin expression on the Ca-P-Ti-coating was compared to pure Ti-disks, whereas our control stainings were made on glass slides, which presumably explains the differences. In conclusion, several studies have been made with various mechanical treatments and surface modifications of HA-coated titanium, and therefore the studies have produced diverse results of the events occurring on the cellular level.

The observation that the focal adhesions on ALD-HA did not stain as brightly and in spot-like fashion as on glass slides could be explained by the very large surface area of the HA coatings. The glass slide is very smooth and dense, leading to the formation of thick concentrated adhesion structures, compared to the rough surface of the HA coating, where the adhesion structure must cover a larger area three-dimensionally. This might create focal adhesions difficult to visualize with immunofluorescence methods. A similar structure develops on bone slices having a rougher saw cut surface, as the vinculin staining on bone slices resembled closely the staining on ALD-HA. The observation of the staining being similar on the bone slice and ALD-HA might indicate that the ALD-HA surface has a huge surface area related to plate-like HA crystals pointing out from the surface. Based on the results of this study, we assume that the HA nanocrystals provide a sufficient nanorough adhesive surface for cell adhesion. In addition, the actin stress fibers were much more numerous on cover glass and bone compared to ALD-HA. These findings are in line with the recent publication of Taniguchi et al. [89], where poorly oriented stress fiber organization was observed on rough zirconia surface indicating the importance of the surface topography.

The MTT test results confirmed that the cell viability on ALD-HA samples was comparable to the viability on cover glasses and bone slices, although on ALD-HA the viability was significantly lower when tested statistically. However, the MTT test results are directly proportional to the number of viable cells on the samples. Therefore, the results indicate that the cell number was lower on ALD-HA samples. The lower cell number is also visible in SEM and immunofluorescence images (Figure 1 and Figure 2). The observation that MC3T3 cells seemed to have higher cell viability on bone compared to cover glasses also results from higher cell numbers on bone, visible in Figure 1 and Figure 2. Bone appears to be the most favorable surface for the MC3T3 attachment, which can be explained by the osteoblast lineage origin of these cells. On plastic and cover glasses MC3T3 cells tend to form sheet-like monolayers [90,91], and a similar morphology has also been observed on bare and ECM protein-coated titanium surfaces [60,63,65,66,92]. Based on the aspect ratios of the cells in this study, we observed that the cells on cover glass were more round compared to the elongated cells on ALD-HA and bone. The morphology of MC3T3 cells on bone has not been studied in detail. Still, concerning osteoblast activity, including proliferation and collagen synthesis, Matsumoto et al. [93] have shown that sintered bone is a more favorable surface for MC3T3 cells compared to glass or HA-related material. This might explain the highest cell viability observed on bone slices in this study.

The morphology of the MC3T3 cells on the bone observed in this study bears a close resemblance to the morphology of the stromal cells from bone marrow. The stromal cell population often termed mesenchymal stem cells, is a heterogeneous cell population present in bone marrow [94,95] that can differentiate into osteoblasts, chondrocytes, adipocytes and fibroblasts [96,97]. Our previous studies showed that the stromal cells form a sheet-like structure on bone slices [98,99], simulating the canopy structure covering the bone remodeling sites in vivo [100]. The canopy is suggested to be formed either from bone lining cells [100] or from the bone marrow sac cells, which are stromal cells located above the bone lining cell layer on the endosteal side of the bone marrow [101,102]. Therefore, it is not surprising that the MC3T3 cells behave in a similar way on bone, as they likewise originate from the stromal cell lineage. As previously mentioned, the bone might be the most favorable substrate for the MC3T3 attachment explaining the results of this study. However, we demonstrate for the first time a sufficient cell adhesion and viability also on a thin HA layer prepared with the ALD method. The results offer new possibilities for developing better implant coatings leading to improved osseointegration and implant survival.

## 5. Conclusions

For the first time, this study demonstrates that osteoblast lineage MC3T3 cells attach to a thin atomic layer deposited HA on Ti substrate with focal adhesions as observed on glass slides and bone. This indicates cellular adhesion to the surface and shows that ALD-HA is biocompatible concerning cell attachment. However, no cell adhesion or morphology changes on FN-coated samples are observed, depicting that the ALD-HA surface is suitable for cellular adhesion without additional ECM protein coating. Cell viability on ALD-HA was comparable to viability on glass slides and bone. In conclusion, these results suggest that ALD-HA is a suitable coating for Ti-implants and can be further developed for obtaining improved implant solutions.

## Data Availability

The data that support the findings of this study are available on request from the corresponding author.
